# Supplementary material for: Membrane procoagulation and N‑terminomics/TAILS profiling in Montreal platelet syndrome kindred with VWF p.V1316M mutation
Source: Commun Med (Lond). 2023 Sep 21;3:125. doi: 10.1038/s43856-023-00354-1 (PMC10514327; doi:10.1038/s43856-023-00354-1)
Supplement: Supplementary file 1 — Reporting Summary [file 43856_2023_354_MOESM1_ESM.pdf]

nature portfolio

Corresponding author(s): Antoine Dufour
Last updated by author(s): Sep 1, 2023

Reporting Summary

Nature Portfolio wishes to improve the reproducibility of the work that we publish. This form provides structure for consistency and transparency in reporting. For further information on Nature Portfolio policies, see our Editorial Policies and the Editorial Policy Checklist.

Statistics

For all statistical analyses, confirm that the following items are present in the figure legend, table legend, main text, or Methods section.

n/a	Confirmed	
☐	☒	The exact sample size (*n*) for each experimental group/condition, given as a discrete number and unit of measurement
☐	☒	A statement on whether measurements were taken from distinct samples or whether the same sample was measured repeatedly
☐	☒	The statistical test(s) used AND whether they are one- or two-sided<br>*Only common tests should be described solely by name; describe more complex techniques in the Methods section.*
☐	☒	A description of all covariates tested
☐	☒	A description of any assumptions or corrections, such as tests of normality and adjustment for multiple comparisons
☐	☒	A full description of the statistical parameters including central tendency (e.g. means) or other basic estimates (e.g. regression coefficient) AND variation (e.g. standard deviation) or associated estimates of uncertainty (e.g. confidence intervals)
☐	☒	For null hypothesis testing, the test statistic (e.g. *F*, *t*, *r*) with confidence intervals, effect sizes, degrees of freedom and *P* value noted<br>*Give P values as exact values whenever suitable.*
☐	☒	For Bayesian analysis, information on the choice of priors and Markov chain Monte Carlo settings
☐	☒	For hierarchical and complex designs, identification of the appropriate level for tests and full reporting of outcomes
☐	☒	Estimates of effect sizes (e.g. Cohen's *d*, Pearson's *r*), indicating how they were calculated

Our web collection on statistics for biologists contains articles on many of the points above.

Software and code

Policy information about availability of computer code

Data collection	There is now a Code Availability supplementary date file that povide information about the sfowtare versions: Nikon NIS-Elements imaging software v1, Xcalibur (version 4.0.21.10), MaxQuant software v.1.6.0.1, STRING.v11 database (https://string-db.org), Metascape v3.5 (https://metascape.org).
Data analysis	Data analysis was performed using Nikon NIS-Elements imaging software v1, Xcalibur (version 4.0.21.10), MaxQuant software v.1.6.0.1, STRING.v11 database (https://string-db.org), Metascape v3.5 (https://metascape.org).

For manuscripts utilizing custom algorithms or software that are central to the research but not yet described in published literature, software must be made available to editors and reviewers. We strongly encourage code deposition in a community repository (e.g. GitHub). See the Nature Portfolio guidelines for submitting code & software for further information.

Data

Policy information about availability of data

All manuscripts must include a data availability statement. This statement should provide the following information, where applicable:

- Accession codes, unique identifiers, or web links for publicly available datasets
- A description of any restrictions on data availability
- For clinical datasets or third party data, please ensure that the statement adheres to our policy

The full proteomics data are publicly available. The data were deposited in PRIDE Archives, accession number: PXD035713 (ProteomeXchange accession:

PXD035713). Project Webpage: http://www.ebi.ac.uk/pride/archive/projects/PXD035713.

Human research participants

Policy information about studies involving human research participants and Sex and Gender in Research.

Reporting on sex and gender	As this is a rare genetic disease, the patient information is well known and has been characterized before. This is the information that was added in the manuscript: "Eight previous studies have reported on the two patients of the present study6,7,10,11,13-16; (Supplementary Table 6). The two 2B-VWDMPS patients in this study are patients L.T.B. (current age 81 years) and her daughter I.B. (age 64) reported previously6,11."
Population characteristics	This is a case study as there are 2 patients with a rare mutation.
Recruitment	This is a case study as there are 2 patients with a rare mutation.
Ethics oversight	Written informed consent was obtained in accordance with the Declaration of Helsinki. Blood samples were obtained from participants under the University of Calgary, Research Ethics Board approval (REB15-0550). All methods were performed in accordance with the Alberta Health Services and The University of Calgary research guidelines and regulations.

Note that full information on the approval of the study protocol must also be provided in the manuscript.

Field-specific reporting

Please select the one below that is the best fit for your research. If you are not sure, read the appropriate sections before making your selection.

☒ Life sciences ☐ Behavioural & social sciences ☐ Ecological, evolutionary & environmental sciences

For a reference copy of the document with all sections, see nature.com/documents/nr-reporting-summary-flat.pdf

Life sciences study design

All studies must disclose on these points even when the disclosure is negative.

Sample size	This is a case study as there are 2 patients with a rare mutation.
Data exclusions	No data was excluded.
Replication	our data was replicated as many times as needed and as much as possible before our samples ran out. As this is a case study, we were only able to get limited amount.
Randomization	Data were randomized when possible.
Blinding	experiments were blinded when possible.

Reporting for specific materials, systems and methods

We require information from authors about some types of materials, experimental systems and methods used in many studies. Here, indicate whether each material, system or method listed is relevant to your study. If you are not sure if a list item applies to your research, read the appropriate section before selecting a response.

Materials & experimental systems

n/a	Involved in the study	
☐	☒	Antibodies
☒	☐	Eukaryotic cell lines
☒	☐	Palaeontology and archaeology
☒	☐	Animals and other organisms
☐	☒	Clinical data
☒	☐	Dual use research of concern

Methods

n/a	Involved in the study	
☒	☐	ChIP-seq
☒	☐	Flow cytometry
☒	☐	MRI-based neuroimaging

Antibodies

Antibodies used	CLIC1 primary antibody from Cell Signaling technology (clone D7D6H, batch #53424); beta actin primary antibody from Abcam (clone and batch ab227387); Thrombin (F-1) primary antibody from Santa Cruz Biotechnology, Inc (Catalog #sc-271449); VWF (C-12) primary antibody from Santa Cruz Biotechnology, Inc (Catalog #sc-365712); Annexin V, Alexa Fluor® 568 conjugate (product code A13202); Alexa Fluor® 488 anti-human CD62P (P-Selectin) Antibody (Catalog #304916).
Validation	All protocols and antibodies were used as per the manufacturer's recommendations and validated before being used for the publication.

Clinical data

Policy information about clinical studies

All manuscripts should comply with the ICMJE guidelines for publication of clinical research and a completed CONSORT checklist must be included with all submissions.

Clinical trial registration	N/A
Study protocol	N/A
Data collection	N/A
Outcomes	N/A
