# Supplementary material for: Membrane procoagulation and N‑terminomics/TAILS profiling in Montreal platelet syndrome kindred with VWF p.V1316M mutation
Source: Commun Med (Lond). 2023 Sep 21;3:125. doi: 10.1038/s43856-023-00354-1 (PMC10514327; doi:10.1038/s43856-023-00354-1)
Supplement: Supplementary file 5 — Supplementary Information [file 43856_2023_354_MOESM5_ESM.pdf]

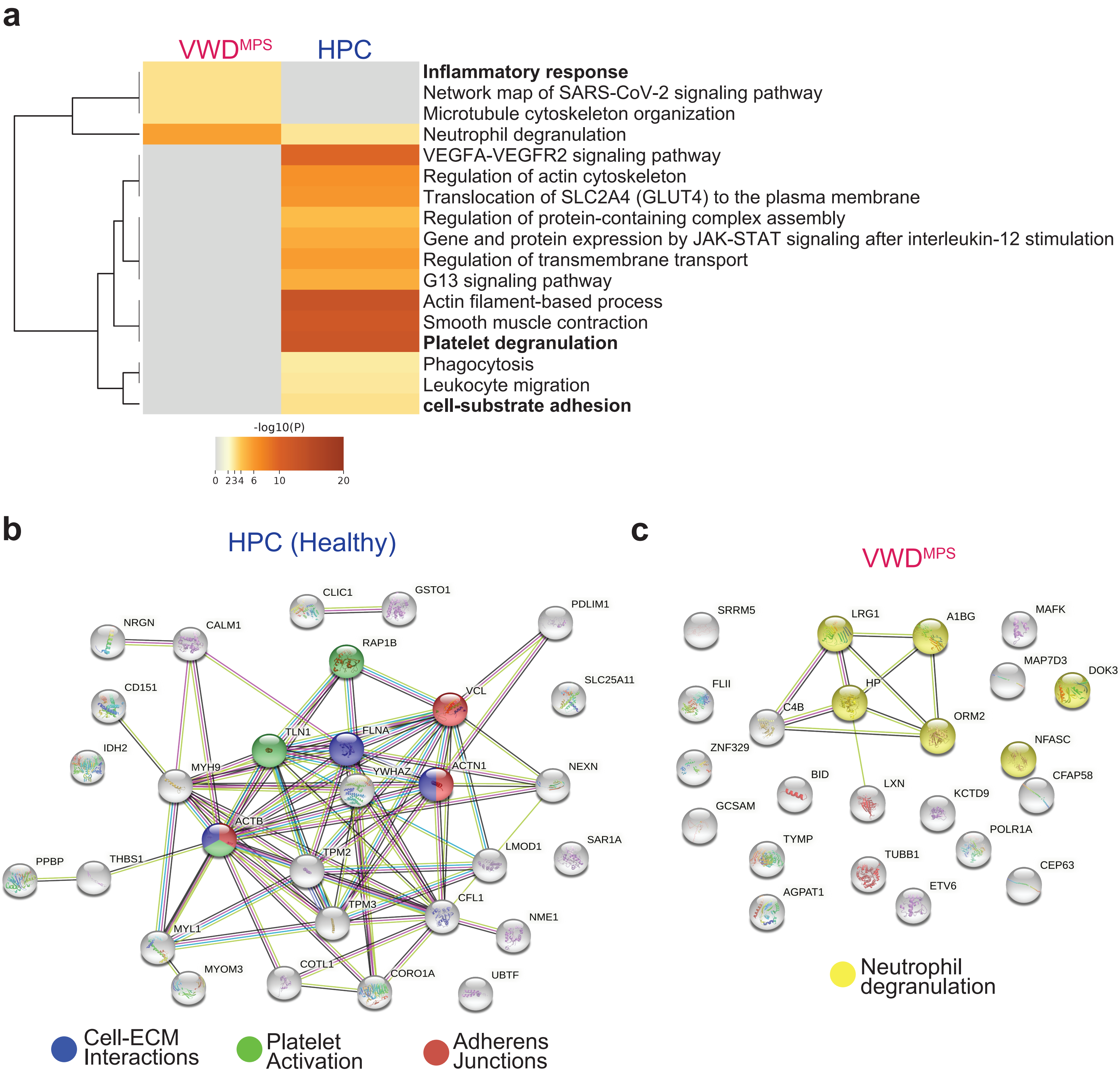

Supplementary Figure 1: **a)** Metascape analysis (www.metascape.org) of significantly changing proteins in healthy (HPC) and 2B-VWD[mps] patients. String-DB pathway analysis (www.string-db.org) of significantly changin proteins in **b)** healthy (HPC) and **c)** 2B-VWDmps patients.

a

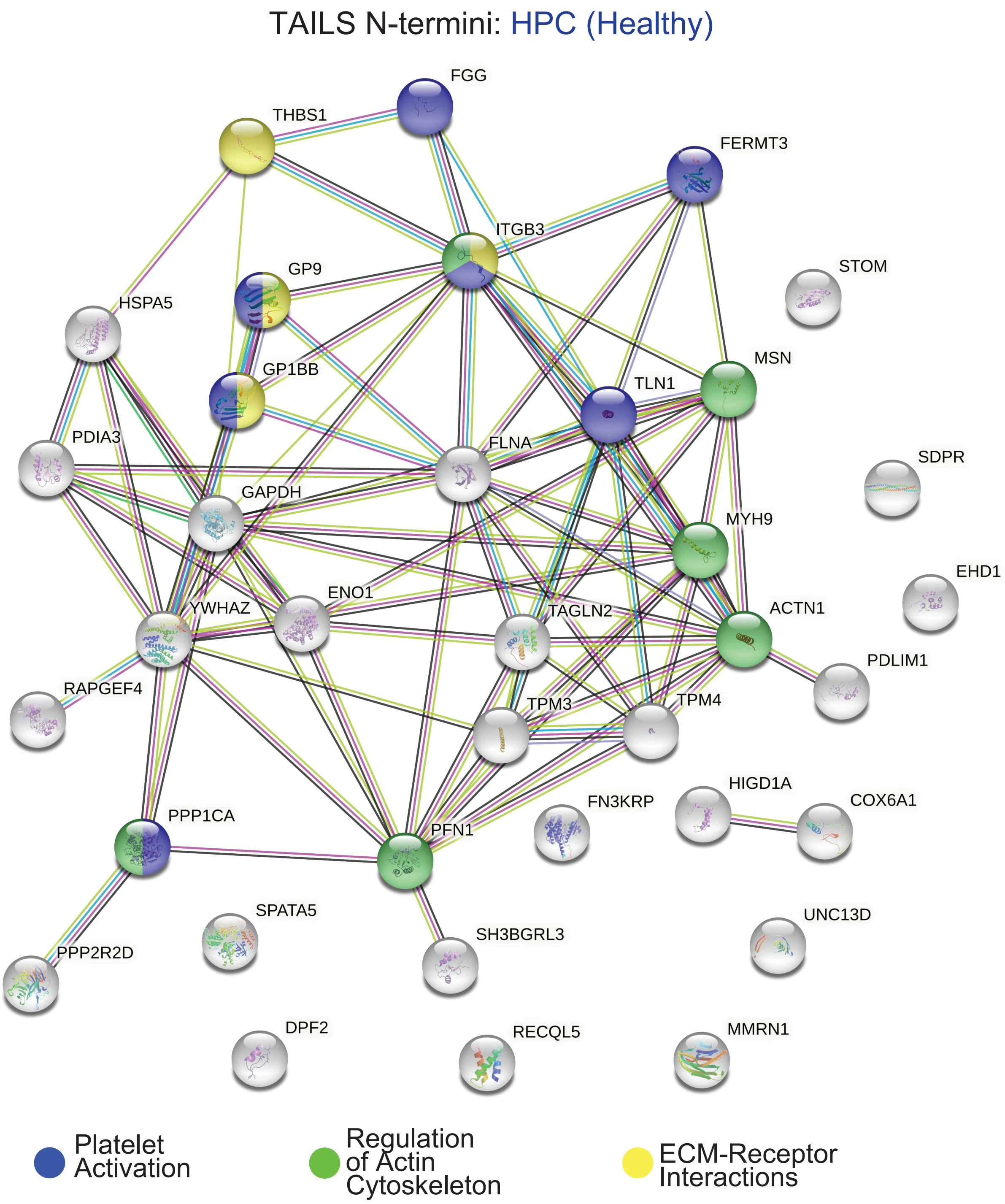

Supplementary Figure 2: **a)** String-DB pathway analysis (www.string-db.org) of signficant N-terimini in healthy (HPC) patients.

a

CLIC1 Ab from Cell signaling, #53424

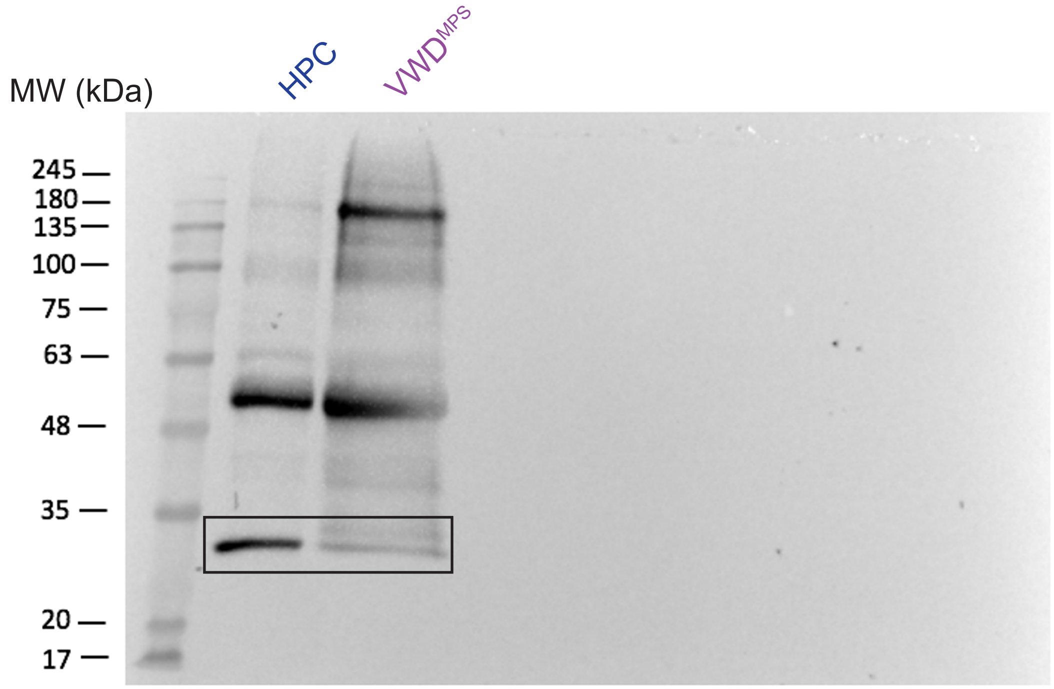

b

Beta-Actin Ab from Abcam, ab227387

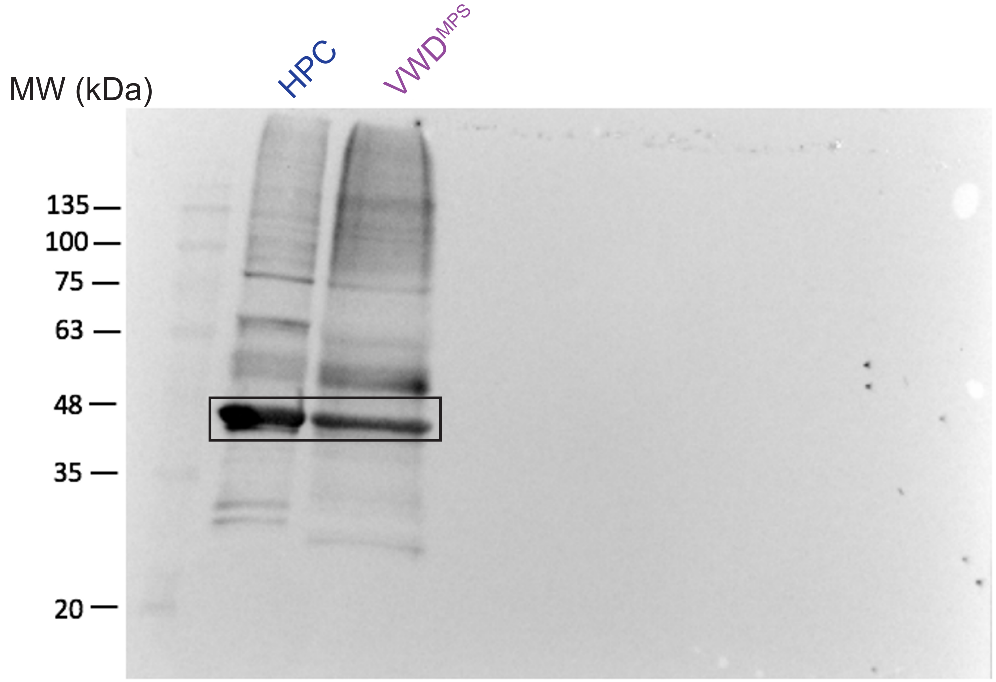

Supplementary Figure 3: Full gel picture of Western blots of **a)** CLIC1, and **b)** beta-actin.
