# Supplementary material for: Membrane procoagulation and N‑terminomics/TAILS profiling in Montreal platelet syndrome kindred with VWF p.V1316M mutation
Source: Commun Med (Lond). 2023 Sep 21;3:125. doi: 10.1038/s43856-023-00354-1 (PMC10514327; doi:10.1038/s43856-023-00354-1)
Supplement: Supplementary file 7 — Description of Additional Supplementary Files [file 43856_2023_354_MOESM7_ESM.pdf]

Description of Additional Supplementary Files

File Name: Supplementary Data 1

Description: List of all previous publications featuring the two patients of the present study.

File Name: Supplementary Data 2

Description: Pre-enrichment TAILS peptides from healthy and 2B-VWD(MPS) patients.

File Name: Supplementary Data 3

Description: Pre-enrichment TAILS proteins from healthy and 2B-VWD(MPS) patients.

File Name: Supplementary Data 4

Description: N-terminomics TAILS peptides from healthy and 2B-VWD(MPS) patients.

File Name: Supplementary Data 5

Description: Proteases and protease inhibitors from healthy and 2B-VWD(MPS) patients.

File Name: Supplementary Data 6

Description: List of major contractile proteins changing in platelets of 2B-VWD(MPS) patients.

File Name: Supplementary Data 7

Description: Source data

File Name: Supplementary Movie 1

Description: Video of control and VWD platelets stained with Annexin-V (red) and P-Selectin (green).
